# Twitch force in human Amyotrophic Lateral Sclerosis

**DOI:** 10.3389/fneur.2025.1590950

**Published:** 2025-05-07

**Authors:** Laura Libonati, Chiara Cambieri, Marco Ceccanti, Federica Moret, Matteo Di Giulio, Eleonora Palma, Maurizio Inghilleri

**Affiliations:** ^1^Department of Human Neurosciences, Rare Neuromuscular Diseases Center, Sapienza University of Rome, Viale Dell’Università, Rome, Italy; ^2^Department of Physiology and Pharmacology, Istituto Pasteur-Fondazione Cenci Bolognetti, University of Rome Sapienza, Rome, Italy; ^3^IRCCS Neuromed, Pozzilli, Italy

**Keywords:** amyotrophic lateral sclerosis, muscle function, electromechanical coupling, disease phenotype, disease progression

## Abstract

**Introduction:**

This study investigated differences in muscle twitch force between slow and fast progressors of amyotrophic lateral sclerosis (ALS) to better understand disease heterogeneity and identify potential biomarkers of disease progression.

**Methods:**

Forty-four ALS patients were classified as slow or fast progressors based on disease progression rates. Electrophysiological assessments, including compound muscle action potential (CMAP) and muscle force measurements, were conducted. Creatine kinase (CK) levels were also evaluated.

**Results:**

Slow progressors demonstrated significantly higher muscle peak force and area under the curve (AUC) compared to fast progressors, reflecting greater muscle strength and endurance. CK levels were also elevated in slow progressors.

**Discussion:**

Despite similar CMA*p* values, slow progressors retained greater muscle strength, possibly due to a reduced degeneration of fast-twitch fibers and compensatory axonal sprouting. These adaptations may preserve muscle function and elevate CK levels, suggesting better muscle integrity in slow progressors.

**Conclusion:**

Muscle function profiles and CK levels are promising indicators of ALS progression. These findings could enhance early detection of disease progression and lead to targeted interventions to preserve muscle function. Further research is needed to validate these results and explore the underlying functional mechanisms of disease heterogeneity.

## Highlights

- *Differentiation of ALS Phenotypes*: ALS slow progressors show greater muscle strength despite similar electrophysiological traits, highlighting phenotype diversity.
- *Insight into ALS Pathogenesis*: Study underlines the muscle’s role in ALS, emphasizing the muscle function & electromechanical coupling’s impact on disease progression.
- *Implications for Therapeutic Interventions*: Linking muscle function to ALS progression can preserve muscle integrity, slow disease progression, and enhance patient outcomes.

## Introduction

Amyotrophic Lateral Sclerosis (ALS) is a fatal adult-onset neurodegenerative disease characterized by progressive degeneration of motor neurons in the brain cortex, brainstem, and spinal cord, leading to muscular weakness, atrophy, respiratory failure, and death within 2–3 years of symptom onset in most cases (1–4). While neuronal dysfunction marks the disease’s initial phase, the mechanisms underlying progression remain poorly understood (5). Clinically, ALS is highly heterogeneous, with variability in affected body regions, the degree of upper and lower motor neuron involvement, and associated behavioral and cognitive changes (6, 7). Disease progression also varies, with patients categorized as fast or slow progressors based on the progression index (PI), calculated as the monthly decline in the ALS Functional Rating Scale-Revised (ALSFRS-R) score from symptom onset (8, 9). However, the factors driving this variability—whether intrinsic or environmental—are not fully elucidated, and reliable biomarkers to predict disease progression remain lacking (10).

Understanding factors that influence disease progression is crucial for developing targeted therapies. Recent research has highlighted the potential role of muscles in the pathophysiological process of ALS, with compensatory mechanisms, such as collateral sprouting of nerve fibers, mitigating clinical effects in the early stages of the disease (11, 12). Muscle strength is determined by a combination of factors, including muscle fiber size and type, as well as neurological components like cortical excitability, neuromuscular junction integrity, electromechanical coupling, and the efficiency of the muscle contraction system (13, 14).

In ALS, motor neuron degeneration leads to progressive muscle atrophy and loss of force. Compound muscle action potential (CMAP), which reflects the sum of motor unit action potentials (MUAPs) generated at motor endplates, decreases as the disease advances (15, 16). Additionally, late-stage ALS is marked by a reduced electromechanical coupling efficiency and variable degeneration of muscle fiber types (17, 18). Despite these changes, there is limited understanding of how these factors differ between fast and slow progressors.

This study aimed to investigate electromechanical coupling efficiency in ALS by focusing on the flexor muscles of the fingers. Specifically, we compared the contractile force evoked by supramaximal stimulation of the median nerve at the elbow with the amplitude of CMAP recorded in the flexor muscles of the fingers. By analyzing differences between fast and slow progressors, we sought to gain insights into the mechanisms underlying disease heterogeneity and progression.

## Materials and methods

We enrolled 44 patients (22 males and 22 females) diagnosed with definite Amyotrophic Lateral Sclerosis (ALS) according to the revised El Escorial criteria (19). Diagnosis was made by experienced neurologists based on clinical and electrophysiological assessments.

Bulbar onset was observed in 10 patients. All patients provided written informed consent before study enrollment. The study complied with the principles of the Declaration of Helsinki and international safety guidelines. Patients were divided into two groups: fast and slow progressors according to the monthly reduction in the ALS Functional Rating Scale (ALSFRS-R) from the onset of symptoms to the day of evaluation.

The ALS Functional Rating Scale-Revised (ALSFRS-R) is a validated 48-point scale measuring physical function in ALS across four domains: bulbar, fine motor, gross motor, and respiratory. Each item is scored from 0 (worst) to 4 (normal), with a maximum score of 48 indicating full function. We used this scale to calculate a Progression Index (PI), which is defined as: PI = (48 - ALSFRS-R at diagnosis) / months from symptom onset to diagnosis. Patients with PI ≤ 0.5 were defined as slow progressors; those with PI > 0.5 were considered fast progressors (20).

For each patient, we concurrently recorded the latency and amplitude of the compound muscle action potential (CMAP) and the force produced at the level of the flexor digitorum profundus muscle, which stimulates supra-maximally the median nerve at the elbow.

This study used a Micromed Myoquick 1,400 EMG machine (Micromed S.p. A., Treviso, Italy). CMAP was recorded using surface electrodes (15 × 20 mm, model DENIS15026, Neuro Dart, Spes Medica, Genoa, Italy). The active electrode was placed on the flexor digitorum profundus muscle belly in the forearm, whereas the reference electrode was placed 10 cm distally, in an electrically neutral region.

**Figure 1:**
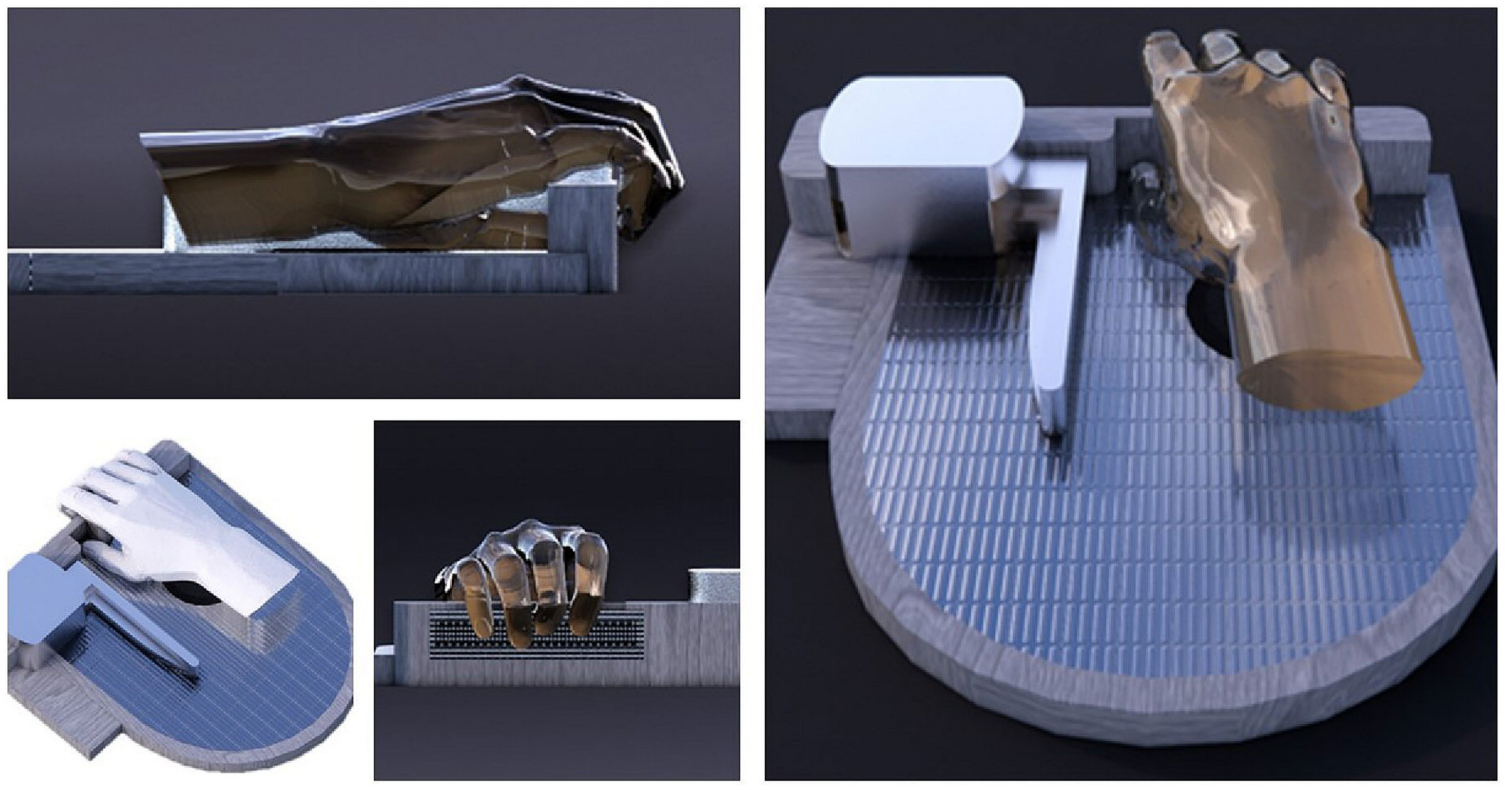
The device: the patient’s hand is placed on a rigid surface while the fingers touch the sensor.

Film Pressure Sensor RP-L-110 Resistive film, configured as a closed Wheatstone bridge, was used to detect and quantify human touch pressure. It is a robust polymer thick film (PTF) device that exhibits a decrease in resistance with increasing force applied to the sensor surface. The device was affixed to another rigid surface, and participants were instructed to place their fingers on this surface to register the touch pressure, as shown in Figure 1. Peak force was defined as the maximum force generated after the supramaximal stimulation of the median nerve at the elbow.

We delivered a single square stimulus with a duration of 200 μs using a constant-current stimulator. The intensity of the stimulus increased steadily until the best response could be obtained. The best motor response had the shortest latency and largest amplitude. Both upper limbs were tested in each participant. For data analysis, we selected the limb with better preserved motor function, as determined by patient history and clinical evaluation, to reduce variability due to asymmetrical progression. We also recorded the creatine kinase (CK) level closest to the day of the visit.

The IBM SPSS Statistics Data version 26 software was used to analyze the data. The Kruskal–Wallis test for independent samples was used to explore group differences. We analyzed the differences between the latency and amplitude of the CMAP and between the amplitude and area of the force developed between the two groups. Mann–Whitney U test for independent samples was used to explore the differences in CK values between the groups. The significance level was set at *p* < 0.05.

## Results

According to the revised El Escorial criteria, a total of 44 patients with a definitive diagnosis of amyotrophic lateral sclerosis (ALS) were enrolled in this study. These patients were subsequently stratified into two groups based on their progression index (PI). The first group consisted of 22 patients (10 males and 12 females), among whom 17 presented with spinal-onset ALS, while the remaining 5 exhibited bulbar-onset ALS. These individuals had a PI of ≤0.5 and were classified as slow progressors. The second group also included 22 patients (12 males and 10 females), with an identical distribution of ALS onset types (17 spinal-onset and 5 bulbar-onset). However, these individuals had a PI greater than 0.5 and were categorized as fast progressors.

In assessing the electrophysiological characteristics of the compound muscle action potential (CMAP), no statistically significant differences were observed between the two groups. The mean CMAP latency was 2.54 ± 0.55 ms in the fast progressor group, compared to 2.43 ± 0.45 ms in the slow progressor group. Similarly, the mean amplitude of the CMAP did not show a substantial variation, with fast progressors exhibiting an amplitude of 10.45 ± 4.45 mV, while slow progressors demonstrated an amplitude of 9.49 ± 4.78 mV. These findings suggest that CMAP characteristics alone may not be sufficient to distinguish between fast and slow disease progression in ALS patients.

However, significant differences were detected in measures of muscle strength and force generation. The amplitude of the peak force was markedly higher in slow progressors (10.5 ± 5.7 Newtons [N]) compared to fast progressors (6.3 ± 5.1 N), with a statistically significant *p*-value of 0.005. Additionally, the area under the curve (AUC) of force production demonstrated a significant discrepancy between the two groups. Slow progressors exhibited an AUC of 6,465 ± 5,300 mV/ms, whereas fast progressors had a considerably lower value of 3,600 ± 3,475 mV/ms (*p* = 0.017). These findings suggest that muscle strength and endurance, as quantified by peak force and AUC, are strongly correlated with disease progression rate in ALS.

Notably, the creatine kinase (CK) levels between the two groups were different. Indeed, CK values were significantly higher in slow progressors (367.3 ± 277.76 U/L) compared to fast progressors (188.2 ± 110.49 U/L), with an extremely significant *p*-value of less than 0.001. This suggests that CK levels may serve as a potential biomarker for disease progression, with elevated CK values being more commonly associated with a slower rate of decline in ALS patients.

A comprehensive summary of these results, including all measured variables, is presented in Table 1. Altogether, these data highlight some key physiological differences between slow and fast progressors, which may offer valuable insights into disease mechanisms and potential therapeutic targets. The observed distinctions in muscle strength, endurance, and CK levels warrant further investigation to explore their potential role in disease monitoring and prognosis.

**Table 1:** Characteristics of the cMAP latency and amplitude, recorded force and CK values of ALS patients, divided into fast and slow progressors.

	FAST progressors	SLOW progressors	*p* value
cMAP			
Latency (ms)	2.542 ± 0.552	2.426 ± 0.448	0.858
Amplitude (mV)	10.45 ± 4.449	9.488 ± 4.778	0.656
FORCE			
Area (mV/ms)	3,600 ± 3,475	6,465 ± 5,300	0.017
Peak force amplitude (N)	6.3 ± 5.1	10.5 ± 5.7	0.005
CK	188.2 ± 110.49	367.3 ± 277.76	<0.001

All data are expressed as mean ± SD.

## Discussion

The pathogenesis of ALS remains unclear, and there are currently two main hypotheses regarding its spread: the dying forward and dying back theories (21). More recent discoveries have highlighted evidence that pathological changes may begin at the level of the nerve terminal or at the neuromuscular junction (NMJ) and then progress upwards, according to the dying back model (22). Muscle tissue has also emerged as a significant factor in ALS, both in its onset and as a potential therapeutic target (11).

**Figure 2:**
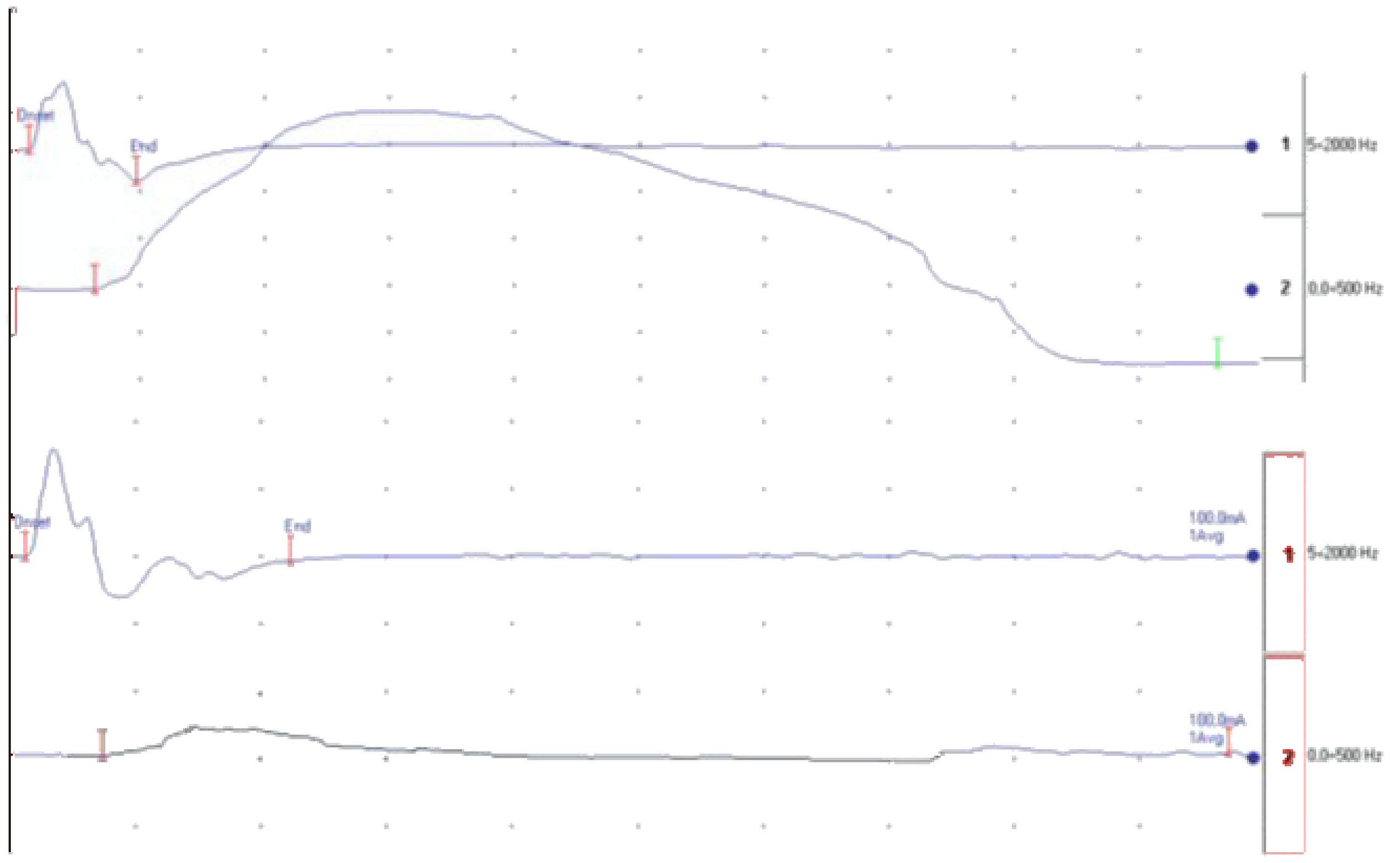
At the top is the recording of the cMAP, and at the bottom is the recording of the force. The first image belongs to a slow progressor patient, while the second one to a fast progressor. Both recordings were obtained from the supramaximal stimulation of the median nerve at the elbow.

In this study, we focused on slow progressors, that are ALS patients who exhibit a low monthly reduction in the ALSFRS-R score. Interestingly, these patients demonstrated ability to develop greater muscle strength despite having the same CMAP values as their faster progressing counterparts (Figure 2).

Muscle contraction is a complex phenomenon that occurs when the action potential propagates along the MNs and reaches the NMJ, resulting in the release of acetylcholine in the synaptic cleft. The binding of acetylcholine to the nicotinic receptor can induce the propagation of an action potential along the sarcolemma with the consequent release of calcium and the electromechanical coupling of the actin and myosin filaments responsible for muscle contraction. Muscular strength development is influenced by factors such as MN firing rate, motor units number and type, and muscle size (23). There are several distinct muscle fibers within muscle tissue that are characterized by different isoform of myosin heavy chain (MyHC) and metabolic activity. Specifically, in human there is one type of slow-twitch fiber (type I) and two types of fast-twitch fibers (type IIa and IIx/d) (24). Feeback et al. (25) reported that the energy for contraction of Type-I (slow-twitch) fibers was mainly supplied by phosphocreatine shuttle, while in Type-II (fast-twitch) fibers was supplied via energy supply pathway due to glycolytic metabolism. In the two types of single muscle fibers, the CK isoenzymes may play different roles in intracellular energy metabolism. It has been reported that fast-twitch fibers have a higher concentration of phosphocreatine than slow-twitch fibers (26). These muscle fibers are sensitive to several factors that cause muscle atrophy and degeneration.

In ALS, muscle atrophy occurs early together with the dismantling of the NMJ (27). The degeneration of MNs in ALS is not homogeneous since different vulnerabilities were observed in different cells. The first cells undergoing degeneration are those that innervate fast-twitch muscle fibers. Slow-twitch muscle fibers are more resistant to degenerative phenomena and, at least in the initial stages, can compensate for the loss of other fibers with a collateral reinnervation (28). Nevertheless, this mechanism is not effective as compared with the degenerative process typical of ALS. Notably, enlargement of the motor unit potential and twitch force (29) was observed, but it is still not clear whether these reorganized motor units have the same characteristics as normal motor units or as those formed in other reinnervation processes. An increase of motor-unit twitch force has been reported in chronically denervated muscles (30) but not in all ALS patients (31). The MN sprouting and the resulting increase of twitch force can compensate for the loss of MNs in the early stages of ALS (24). Slow-twitch fibers can sustain muscle force for long time, although they are not capable of generating considerable force as it happens with fast twitch fibers (24).

The NMJs innervated by fast-fatigable MNs degenerate rapidly, followed by fast-fatigue-resistant MNs and, finally by slow MNs (32). The number of motor units declines in parallel with muscular strength, and the muscle fibers in the remaining motor units, undergo hypertrophy as a compensatory mechanism.

In this study, an increased force was predominantly found in patients whose disease progression was slightly slower. The corresponding CMAP values were similar among the groups, indicating that the collateral axonal sprouting process played a role in the compensatory mechanism. The CMAP can serve as a surrogate measure of the number of functional motor units because it reflects denervation and reinnervation processes (33). However, in a rapidly evolving disease, such as ALS, in which reinnervation is not entirely effective, the amplitude of CMAP tends to decrease over time (34). However, the CMAP amplitude can be used roughly to assess disease progression although its accuracy may be compromised by reinnervation (35).

We identified a notable disparity in the peak force amplitude between slow and fast progressors, with slow progressors exhibiting a higher peak. In addition, there was a significant difference in the area under the curve (AUC) for muscle contraction, with slow progressors exhibiting a higher AUC. These findings suggest that slow progressors can maintain a superior muscle function, as evidenced by the elevated peak force and AUC. The peak force reflects the maximum muscle strength that a muscle can exert during contraction, whereas the AUC represents the total power output during muscle contraction. Measuring peak force in ALS can help to evaluate muscle strength preservation or decline due to motor neuron degeneration that leads to muscle weakness and atrophy. Therefore, the elevated values of peak force for slow progressors suggest a better preserved muscle function capable of sustained contraction. Additionally, a higher AUC indicates that the muscle can generate strong contractions and sustain them over the time, which is essential for muscle endurance. In ALS patients, this implies not only strong muscles but also a sustained function, which is potentially associated with slower disease progression. This may contribute to a slower progression of muscle weakness and atrophy.

In fast progressors, the pronounced degeneration of fast-twitching fibers, despite the reinnervation of slow-twitching fibers, leads to a lower muscle force and CK levels. As these fibers degenerate, the muscle’s ability to produce force is diminished, thereby affecting the overall muscle function and strength. Indeed, the slow-twitching fibers, even when reinnervated, cannot fully compensate for the loss of fast-twitching fibers in terms of the force output and maintaining high CK levels (36, 37). Mechanisms such as the activation of specific signaling pathways (e.g., FoxO1-related pathways) (36) predominantly affect fast-twitch fibers, leading to their selective atrophy. On the other hand, slow progressors exhibit less degeneration of fast-twitching fibers, which allows them to maintain higher muscle force and CK levels. This could be due to a greater resistance of fast-twitching fibers to degeneration or more effective compensatory mechanisms that preserve muscle integrity (38). The difference in creatine kinase (CK) production in fast-twitch muscles could be attributed to the greater force generated by these muscles. Fast-twitch muscles, which are known for producing more force and are associated with rapid and powerful muscle contractions, might require higher CK activity to support rapid energy replenishment during intense exercise (39). Muscle-specific creatine kinase (CK-M) is crucial for the reversible transfer of high-energy phosphate from creatine phosphate (CrP) to ADP, a process vital for rapid ATP regeneration during intense muscle activity (40). Therefore, increased CK-M activity in fast-twitch muscles may be linked to their efficient energy-generation capacity during intense muscle contractions.

In addition, there is a weaker electromechanical coupling in ALS patients compared with controls, particularly affecting the fast-contracting motor units. Prolonged fasciculation electromechanical latency in ALS muscles indicates an impairment in the excitation-contraction coupling mechanism, which contributes to muscle weakness (13). We identified novel biomarkers of disease progression to further understand the mechanisms underlying muscle dysfunction in ALS. Furthermore, differences in the electromechanical coupling and synchronous firing of motor units in ALS patients and healthy individuals underline the complexity of ALS pathophysiology. A weaker electromechanical coupling, particularly in fast-contracting motor units, highlights the role of muscle function and its alterations in disease progression (8).

Growing evidence suggest that other non-nerve cells, such as glial cells (41) and muscle cells (42), can contribute to the damage or preservation of MNs. In a previous study it was shown that in animal models and in humans with a better muscle metabolic reserve expressed by elevated CK values, disease progression was much slower (28). Higher CK levels in slow progressors might indicate a greater muscle mass or enhanced muscle integrity, potentially associated with a slower disease progression rate. Because of its heterogeneity, ALS presents distinctive phenotypes, including onset pattern, predominance of upper or lower MN involvement, and disease progression rate, which remain poorly understood. These different characteristics suggest the presence of different underlying molecular processes. However, a clear correlation between neuropathological phenomena and different phenotypes is not yet evident. Our findings deepen the understanding of the variability observed in ALS progression and underline the importance of assessing other physiological parameters rather than conventional electrophysiological measures. Identification of distinct muscle force profiles and elevated CK levels in slow progressors may serve as potential biomarkers for predicting disease progression and assessing treatment effectiveness. Notably, it has been suggested that muscle-specific factors, as SIRT1 overexpression, can affect the fiber type composition and potentially improving the pathophysiology of muscle-related conditions. In fact, SIRT1 promotes a shift toward slow-twitch fibers, potentially affecting disease progression and muscle function (43).

It is important to acknowledge several limitations of the study that may impact the interpretation of the results. Firstly, the sample size was relatively small, which may limit the generalizability of the findings. Secondly, the cross-sectional study design precludes the establishment of causal relationships between the observed parameters and disease progression. Further longitudinal studies with larger participant groups are needed to confirm these findings and explore additional factors influencing ALS progression. Unfortunately, respiratory function data (e.g., FVC or SNIP values) were not available for all participants at the time of evaluation and were thus excluded from this analysis. Future studies should aim to integrate these data to further explore their relationship with muscle function and disease progression this study also opens potential implications for rehabilitation strategies. The preservation of muscle strength in slow progressors suggests that early targeted rehabilitation programs aimed at maintaining muscle integrity could be particularly beneficial in this subgroup. Functional exercise and neuromuscular stimulation might help delay functional decline.

## Conclusion

The significant differences in peak force and AUC in slow and fast progressors suggest that slow progressors retain a better muscle function. Higher CK values in slow progressors may reflect greater muscle mass or integrity, potentially contributing to slower ALS progression in these patients. These findings could provide new insights into the pathogenesis of ALS, predict the disease progression, and develop new targeted therapies aimed at preserving muscle function.

## Data Availability

The raw data supporting the conclusions of this article will be made available by the authors, without undue reservation.
